# Supplementary material for: Acceptability of early childhood obesity prediction models to New Zealand families
Source: PLoS One. 2019 Dec 2;14(12):e0225212. doi: 10.1371/journal.pone.0225212 (PMC6886750; doi:10.1371/journal.pone.0225212)
Supplement: S1 File — (DOCX) [file pone.0225212.s001.docx]

S1 File

**Survey questions on caregivers' views of early childhood obesity prediction models.**

There are ways of working out if a baby is likely to put on too much weight by the time they start school.

We would like to hear from parents and caregivers whether it would be useful to know this information, and what help would be useful to parents if this was the case.

**How concerned would you be if you thought your child was gaining too much weight?**

- Very concerned
- A bit concerned
- Unsure
- Not concerned at all

**Have you had any concerns recently about your child's weight gain?**

- Yes - my child has been putting on too much weight
- Yes - my child isn't putting on enough weight
- No - my child's weight gain has been fine

**Do you have any concerns about your own weight?**

- Often
- Sometimes
- Occasionally
- Never
- Prefer not to answer

**How much control do you think caregivers/parents have over their child's weight?**

- Total control
- A lot of control
- Some control
- Very little control
- No control

**We are interested in how you as a parent or caregiver would like to be given information about your child's weight. For example, at your Well Child check in their first 6 months of life, the Well Child visitor could calculate if your baby has a greater chance of putting on too much weight by the time they start school.**

**With this in mind, please answer the following questions.**

**Would you like to know this information?**

- Definitely yes
- Probably yes
- Maybe
- Probably not
- Definitely not

**How would you feel if you were told your baby was at a greater risk of gaining too much weight when they are older?
Please choose the 3 strongest feelings you think you would experience.**

- Fine
- Worried
- Upset
- Relieved
- Not concerned
- Angry
- Offended
- I wouldn't believe the information

**What would be your preferred way of receiving this information?**

- Face to face
- Email/letter
- Telephone
- Text message
- None of the above (I do not want to hear about it)

**Who would be best to discuss this information with you, what it means, and what changes might be helpful for you and your whānau?**

- Tamariki Ora/Plunket nurse
- Community nurse
- Family doctor or nurse
- Nobody, I wouldn't want to hear about it
- Family/friends
- Other, please specify ________________________________________________

**What important qualities should this healthcare professional have?
Please choose all that apply.**

- Familiar (known to me)
- Trustworthy
- Knowledgeable
- Caring
- Non-judgmental
- Takes time to listen and provide helpful advice
- Same culture/ethnicity as me
- Same gender as me

**When do you think is the best time/stage to receive this information?**

- Before the baby is born
- When the baby is born
- When the baby starts eating solid foods
- When baby starts walking
- When the child is older
- Never

**What could be bad about receiving this information?
Please choose up to 3 that are most important to you.**

- It could make me feel guilty
- It could put parents under more pressure
- I wouldn't know what to do anyway, so what's the point?
- People could judge me
- It could put pressure on the child
- There wouldn't be anything bad about receiving this information
- Other, please specify ________________________________________________

**If you were told your baby had a greater chance of gaining too much weight later in childhood, please rate the statements below in terms of how useful they would be to help your baby be healthy

(0= not helpful at all, 5= very helpful)**

| Learning to recognize when my baby is full | 0 | 1 | 2 | 3 | 4 | 5 |
| --- | --- | --- | --- | --- | --- | --- |
| Learning when to introduce solid foods | 0 | 1 | 2 | 3 | 4 | 5 |
| Learning about nutritious food choices | 0 | 1 | 2 | 3 | 4 | 5 |
| Breastfeeding support | 0 | 1 | 2 | 3 | 4 | 5 |
| Learning how to help my baby be more active | 0 | 1 | 2 | 3 | 4 | 5 |
| Learning to understand why my baby is crying | 0 | 1 | 2 | 3 | 4 | 5 |
| Learning about screen time and my baby | 0 | 1 | 2 | 3 | 4 | 5 |
| Getting my baby into a good sleep routine | 0 | 1 | 2 | 3 | 4 | 5 |
| Having support from healthcare professionals | 0 | 1 | 2 | 3 | 4 | 5 |
| If nutritious food was cheaper | 0 | 1 | 2 | 3 | 4 | 5 |
| Having time to prepare nutritious food | 0 | 1 | 2 | 3 | 4 | 5 |
| Having support from family/friends | 0 | 1 | 2 | 3 | 4 | 5 |
