# Supplementary material for: Acceptability of early childhood obesity prediction models to New Zealand families
Source: PLoS One. 2019 Dec 2;14(12):e0225212. doi: 10.1371/journal.pone.0225212 (PMC6886750; doi:10.1371/journal.pone.0225212)
Supplement: S2 File — (DOCX) [file pone.0225212.s002.docx]

S2 File

**Results from a general linear model examining the associations between caregiver's demographic characteristics and their level of acceptance of the obesity prediction model information.**

**Lower scores represent greater acceptance of the prediction information.**

**Parameter estimates**

| **Caregivers' characteristics and levels** | **β (95% CI)** | **p-value** |
| --- | --- | --- |
| Intercept | 2.015 (1.609, 2.421) | <0.0001 |
| Ethnicity (European) | 0.367 (0.121, 0.613) | 0.003 |
| Ethnicity (Māori) | 0.430 (0.157, 0.703) | 0.002 |
| Ethnicity (Pacific) | 0.346 (0.013, 0.680) | 0.042 |
| Ethnicity (Asian) | – |  |
| University education (below university) | 0.045 (-0.085, 0.176) | 0.49 |
| University education (university) | – |  |
| IMD (less deprived) | -0.108 (-0.233, 0.018) | 0.09 |
| IMD (more deprived) | – |  |
| Type (parent) | -0.028 (-0.361, 0.304) | 0.87 |
| Type (grandparent) | -0.459 (-0.847, -0.071) | 0.021 |
| Type (other caregiver) | – |  |
| Sex (male) | -0.281 (-0.476, -0.085) | 0.005 |
| Sex (female) | – |  |

**Estimated marginal means (adjusted means)**

| **Caregivers' characteristics** | **Levels** | **Mean (95% CI)** |
| --- | --- | --- |
| Caregiver ethnicity | European | 2.05 (1.89, 2.21) |
|  | Māori | 2.11 (1.93, 2.29) |
|  | Pacific | 2.03 (1.76, 2.30) |
|  | Asian | 1.68 (1.42, 1.95) |
| Sex | Males | 1.83 (1.61, 2.04) |
|  | Females | 2.11 (1.95, 2.26) |
| University education | Below university | 1.99 (1.83, 2.15) |
|  | University | 1.94 (1.76, 2.12) |
| Type | Parent | 2.10 (1.99, 2.22) |
|  | Grandparent | 1.67 (1.43, 1.91) |
|  | Other caregiver | 2.13 (1.80, 2.46) |
| IMD | Less deprived | 1.91 (1.74, 2.09) |
|  | More deprived | 2.02 (1.85, 2.19) |

**Pairwise comparisons within caregivers' characteristics**

| **Caregivers' characteristics** | **Pairwise comparisons** | **Mean (95% CI)** | **P-value** |
| --- | --- | --- | --- |
| Ethnicity | European vs Māori | -0.06 (-0.22, 0.09) | 0.42 |
|  | European vs Pacific | 0.02 (-0.23, 0.27) | 0.87 |
|  | European vs Asian | 0.37 (0.12, 0.61) | 0.003 |
|  | Māori vs Pacific | 0.08 (-0.17, 0.34) | 0.53 |
|  | Māori vs Asian | 0.43 (0.16, 0.70) | 0.002 |
|  | Pacific vs Asian | 0.35 (0.01, 0.68) | 0.042 |
| Sex | Male vs female | -0.28 (-0.48, -0.09) | 0.005 |
| University education | Below university vs university | 0.05 (-0.09, 0.18) | 0.50 |
| Type | Parent vs grandparent | 0.43 (0.21, 0.65) | <0.001 |
|  | Parent vs other caregiver | -0.03 (-0.36, 0.30) | 0.87 |
|  | Grandparent vs other caregiver | -0.46 (-0.85, -0.07) | 0.021 |
