# Supplementary material for: Acceptability of early childhood obesity prediction models to New Zealand families
Source: PLoS One. 2019 Dec 2;14(12):e0225212. doi: 10.1371/journal.pone.0225212 (PMC6886750; doi:10.1371/journal.pone.0225212)
Supplement: Attachment — Submitted filename: PLOSONE review.docx [file pone.0225212.s003.docx]

PLOSONE review:

Abstract:

1. Lines 34-35: I would remove this sentence (in this form) from the abstract. Rather say (in the results, perhaps) that there was representation across all ethnicities and levels of education
2. Lines 42-43: To what were males, Asians and grandparents more receptive? This sentence is a bit vague and needs %s to be more meaningful.

Intro:

1. There is mention of different ethnicities in the abstract, but little mention of how this is relevant in the introduction. Is there data from NZ that suggests that children under 5 of differing ethnicities differ in terms of overweight/obesity? Are ethnically-different parents different as well (in terms of their own overweight/obesity, as well as education)?

Methods:

1. It would be useful to add stats regarding internet use and access in NZ. This is available through the world bank (<https://data.worldbank.org/indicator/IT.NET.USER.ZS?locations=NZ>) and may help state your case for using a survey that is distributed through social media.
2. Line 123-124: Did parents indicate which of their <5 child they chose? Is there potentially some bias in this?

Results:

1. Lines 220-221: The sentence that explains overweight and obesity could be reworded to be clearer, and the insertion of the number of children would be helpful. The sentence could read as follows: n=?(%) were classified as overweight/obese. Of these children, n=?(%) were obese. Otherwise, it may be easier to split the numbers (X were overweight, Y were obese).

Discussion:

1. Lines 291-294: This information would fit well in the introduction (as per previous comment)

Strengths/Limitations:

1. Lines 395-397: The sentence starting with “Women living with overweight or obesity…” seems misplaced and doesn’t add value in this section.
